# Modeling and simulation of an effectual triangular slotted UWB flexible antenna for breast cancer detection and healthcare monitoring

**DOI:** 10.1371/journal.pone.0320806

**Published:** 2025-04-17

**Authors:** Aparna Singh, R. K. Dwivedi, Vinod Kumar Singh, Manish Sharma, Kanhaiya Sharma, Bulent Yilmaz

**Affiliations:** 1 Department of Physics, School of Basic Sciences, Chhatrapati Shahu Ji Maharaj University, Kanpur, Uttar Pradesh, India; 2 Department of Physics, School of Basic Sciences, Chhatrapati Shahu Ji Maharaj University, Kanpur, Uttar Pradesh, India; 3 Department of Electrical Engineering, S.R. Group of Institutions, Jhansi, Uttar Pradesh, India; 4 Chitkara University Institute of Engineering and Technology, Chitkara University, Chandigarh, Punjab, India; 5 Department of Computer Science & Engineering, Symbiosis International (Deemed University), Pune, Maharashtra, India; 6 GUST Engineering and Applied Innovation Research Center (GEAR), Electrical and Computer Engineering Department, Gulf University for Science and Technology, Hawally, Kuwait; Parul University, INDIA

## Abstract

With refinements in electromagnetics, diverse medical applications have evolved to detect diseases efficaciously. Breast cancer, a dominant cause of mortality among women worldwide, necessitates early diagnosis and screening for timely medical intervention. This research establishes the design, simulation, and analysis of an advanced triangular slotted circular flexible Ultra-wideband (UWB) antenna optimized for breast cancer detection and healthcare monitoring. The proposed antenna employs an extensive frequency range of 2.95 GHz to 24.2 GHz, accomplishing an impressive impedance bandwidth of 156%. It authenticates directional and omnidirectional radiation patterns with compact dimensions of 46.3 × 52.6 × 1.076 mm³. Key aspects divulge a resonance frequency at 14.35 GHz with a significant input reflection coefficient of −37.8 dB. The antenna achieves a peak gain of 3.16 dB at 5.8 GHz, with efficiencies of 59.56% and 66.88% at 5.8 GHz and 4.48 GHz, respectively. A meticulous case study involving SAR evaluation confirms the antenna’s safe exposure levels. For a flat human phantom, SAR values are 0.774 W/kg at 13.5 GHz and 0.712 W/kg at 14.35 GHz for 10 gm of tissue. For the breast phantom model, SAR values are 0.201 W/kg at 11.4 GHz and 0.152 W/kg at 14.35 GHz for 10 gm of tissue. Besides that, the antenna’s flexible design promises an excellent execution under several bending conditions, making it ideal for wearable applications. These findings establish the antenna as an efficient solution for breast cancer detection and healthcare monitoring, combining safety, flexibility, and the aptness to ameliorate early diagnosis while lowering mortality rates. Wearable antennas are pivotal for advanced healthcare applications. This section presents the literature and discusses the work related to flexible UWB antenna designed for breast cancer detection and healthcare monitoring, tackling challenges in early diagnosis and patient care.

## 1. Introduction

The rapid advancement of Wireless Body Area Networks (WBANs) has revolutionized wearable technology, with applications spanning medicine, defense, emergency services, and consumer electronics. WBANs consist of bio-sensors, motion sensors, and wireless antennas, facilitating body-centric wireless communication, categorized into on-body and off-body systems. On-body systems feature sensor nodes with antennas mounted on the body for monitoring biological signals [1–3]. Wearable devices, including smartwatches and bright clothing by brands like Apple and Nike, have enabled the tracking of medically relevant data. Information and Communication Technology (ICT) and e-health solutions connect urban doctors to remote patients, addressing chronic diseases [4,5]. Wearable antennas are gaining attention for healthcare monitoring and breast cancer detection, a disease that accounted for over 2.26 million new cases globally in 2020, per WHO data, and remains a leading cause of death among women [6–8]. Ultra-wideband (UWB) antennas are ideal for medical imaging due to their compact size, flexibility, and high diagnostic accuracy. Operating between 3.1 GHz and 10.6 GHz, UWB antennas enable deeper tissue penetration, which is critical for early breast cancer detection and other biomedical applications [9,10]. They must be lightweight and conformal and maintain performance under bending and stretching. Recent antenna designs with slots and unconventional geometries enhance impedance bandwidth and radiation efficiency, [11]. Safety is paramount, with SAR values kept low to minimize thermal effects from RF energy absorption. Optimized UWB antennas not only meet these safety standards but also deliver high efficiency for wearable health monitoring [12–16]. Microwave imaging, a non-invasive and cost-effective alternative to traditional methods like MRI or PET, leverages the dielectric property contrast between normal and malignant tissues for breast tumor detection, offering significant promise in early diagnosis without the risks of ionizing radiation [17].

Microwave imaging offers a cost-effective, non-invasive, and radiation-free approach for detecting abnormalities in vivo, enabling real-time, portable applications suitable for seamless integration with mobile health systems. By sending RF signals into tissues and analyzing scattered waves, 3D images are generated, leveraging differences in dielectric constants (ℇr) between healthy and pathological tissues to detect even minor lesions [18]. Unlike X-ray mammography, this method avoids compression and harmful radiation, enabling frequent image captures for early detection. Microwave hyperthermia complements imaging by treating breast tumors through targeted electromagnetic energy, offering a painless, cost-effective alternative to surgery. It has proven effective in achieving tumor necrosis, particularly when combined with radiotherapy or chemotherapy. Researchers advocate integrating imaging and treatment systems to streamline processes and enhance patient care [19]. Differential dielectric properties between normal and cancerous breast tissues (1:2.3 to 1:10) underpin microwave sensing's effectiveness. Advanced antennas enhance microwave detection, including horn antennas, CPW designs; patch antennas, and textile-based spiral discs. These developments, particularly in flexible and wearable antenna systems, promise improved cancer detection and treatment efficiency . Abdelghany et al. present a compact UWB antenna (77 ×  40 ×  1.6 mm^3^) for breast cancer detection, covering 3.1–10.6 GHz, achieving a peak gain of 4.1 dBi and 85% efficiency. SAR levels at the left, correct head and stomach vary across 2.45, 3.5, and 5.8 GHz, demonstrating safe exposure. Mahmood et al. designed a 60 ×  50 ×  0.7 mm³ microstrip antenna for WBAN and breast cancer detection, while Srinivasan et al.] introduced a textile antenna using jeans substrate (ℇr =  1.7) for the 2.4 GHz ISM band with input reflection coefficient>  −35 dB and a gain of 145.3 dB. D. N. Elsheakh et al. [20] developed monopole and microstrip antennas for tumor detection operating within 2.2–8 GHz and 2.4 GHz for ISM band applications. Despite advancements, bandwidth restrictions, and incomplete SAR validations highlight areas for improvement. Addressing these gaps, the proposed triangular-slotted circular UWB flexible antenna resonates from 2.95–24.2 GHz with a peak at 14.35 GHz, offering 156% impedance bandwidth, a 3.16 dB gain, and dimensions of 46.3 ×  52.6 ×  1.076 mm³. It demonstrates directional and omnidirectional patterns, excellent bending performance, and low SAR values (0.712 W/kg for flat and 0.152 W/kg for 10 gm tissue models), proving effective for non-invasive diagnostics and remote healthcare monitoring. The study details the antenna design, material selection, geometry, parametric analysis, and performance assessment. Results include surface current, radiation patterns, and SAR studies, concluding with a discussing of future potential in medical diagnostics.

This section details the methodology employed in developing the proposed wearable UWB antenna, highlighting key formulas to ensure optimal performance of the presented antenna.

## 2. Methodology

Designing antennas requires assessing the electromagnetic properties of textile substrates, as changes in substrate properties affect dimensional geometry and resonance frequencies]. Innovative fabrics integrated with electronic components enable advanced functionalities like e-textiles and tracking garments, with embedded antennas providing wireless features without discomfort [21,22]. Materials such as cotton, polyester, jeans, leather, and felt serve as substrates for flexible antennas, with their dielectric constants influencing radiation efficiency. Higher dielectric constants reduce antenna size and decrease efficiency, making compact and practical designs crucial for WBAN applications [23]. Traditional rigid materials are unsuitable for wearables, which benefit from electro-textiles that adapt to stretching and deformation. The performance of wearable antennas depends on the properties of conductive (radiating element) and non-conductive (substrate) materials, selected based on dielectric characteristics, durability, and electrical conductivity. Equation 1 can estimate the material's conductivity.


$$\sigma =\frac{1}{\rho^{\tau }}$$ (1)

Where σ is the material's conductivity, ρ is the surface resistivity, and τ is the thickness of the material []. Fabricating a miniaturized flexible UWB antenna for wearable implants requires low-loss tangent materials with a high dielectric constant to match breast permittivity, enhance coupling, and align simulation with measurement results [24]. The proposed antenna utilizes jeans textile as a substrate, an adhesive copper sheet for the ground, and a patch element. Table 1 outlines the material properties.

**Table 1:** Characteristics of textile material.

Textile Material	Value
Jeans Thickness	1 mm
Measured Relative Permittivity	1.7
Loss Tangent	0.025

Wearable technologies integrate electronic devices and intelligent sensors like antennas directly on the body for continuous health monitoring with minimal energy use. For breast monitoring, wearable microwave sensing and imaging systems offer a cost-effective, non-ionizing, and passive solution for long-term disease detection. The proposed system provides a comfortable textile-based wearable option for breast cancer screening, particularly for young women with dense breast tissue, using microwave imaging as an alternative to X-ray mammography. Material selection is based on dielectric constant measurements, including conductive and substrate materials. High conductivity and low resistivity are essential to minimize losses and optimize power radiation and reception [25]. The material selection and the antenna design were finalized by evaluating the length and width using different formulas mentioned in equations 2, 3, 4, 5, 6, 7, and 8. Finally, the prepared design was further simulated and optimized by CST software. After that, the design of the flat phantom and the breast phantom model is ready with the desired parameters. The vector network analyzer (VNA) is used to measure the real-time performance of the fabricated antenna design, which has yielded satisfactory results in terms of the desired parameters. The calculation of the length (L) and width (W) of the patch, ground size (LG & WG), and the dielectric value is completed by equations 2, 3, 4, 5 and 6.


$$L=\frac{C}{2_{f_{r}}\surd \varepsilon_{eff}}-2\Delta L$$ (2)

$$W=\frac{c}{2f_{r}}\surd \frac{2}{\varepsilon_{r}+1}$$ (3)

$${\text{L}}_{\text{G}}\text{= L+ 6hs}$$ (4)

$${\text{W}}_{\text{G}}\text{ = W + 6hs}$$ (5)

$$\varepsilon_{reff}=0.5\left(\varepsilon_{r}+1\right)+0.5\left(\varepsilon_{r}-1\right){\left(1+\frac{12h_{s}}{W}\right)}^{-0.5}$$ (6)

The extension of the length ∆ L is calculated by Equation 7. Furthermore, equation 8 gives the formula for calculating the radius of the patch.


$$\frac{\Delta L}{h_{s}}=0.412\frac{\left(\varepsilon_{reff}+0.3\right)\left(\frac{W}{h_{s}}+0.264\right)}{\left(\varepsilon_{reff}-0.258\right)\left(\frac{W}{h_{s}}+0.8\right)}$$ (7)

$$R=\frac{87.94}{f_{r}\surd \varepsilon_{r}}$$ (8)

Where L and W are the length and width of the patch as well as the substrate, c is the speed of light, L_G_ and W_G_ are the length and width of the ground, h_s_ is the height of the substrate, and R is the radius of the patch measured in mm, f_r_ is the resonance frequency, and ℇ_r_ is the permittivity.

This section contours the geometrical design, simulation, and validation of the proposed wearable UWB antenna for effective breast cancer detection and healthcare monitoring.

## 3. Antenna design

### 3.1 Geometrical configuration of the presented antenna

Effective transmission of signals during microwave imaging requires the appropriate antenna design. Previous wearable UWB antennas suffer from low resolution, low bandwidth, high SAR values, and significant size issues. A wearable antenna should have broad bandwidth, low SAR, and be compact with unique characteristics for early breast tumor detection. Microstrips patch antennas with conformal designs, i.e., low cost and easy manufacturing, are extensively used. They comprise a conductive ground layer, dielectric, and conducting patch. While generally narrowband in design, substrate thickness can be varied to tune bandwidth. It is also possible to enhance the performance of this antenna by incorporating notches and slots on patches for wide bandwidth applications [26,27]. A jeans material (ℇr =  1.7 and tanδ =  0.025) is integrated at the center of two copper patches as a substrate, and one patch functions like a radiator while the bottom acts like a ground plane. This configuration increases the bandwidth and decreases surface wave losses. Moreover, a circular slot and partial ground method are used to overcome the bandwidth limitations. Therefore, the circular shape was preferred for the antenna due to its compact area and volume contrasted to other geometries, which facilitates higher bandwidth. The incorporation of a triangular slot in the circular structure amplifies impedance matching and supports ultra-wideband (UWB) operation. This design insures effective resonance at the desired frequencies with fundamental input reflection coefficient and stable radiation patterns. Additionally, the shape enables omnidirectional and directional patterns, making it ideal for wearable applications, including healthcare monitoring and breast cancer detection [28–30].

**Fig 1:**
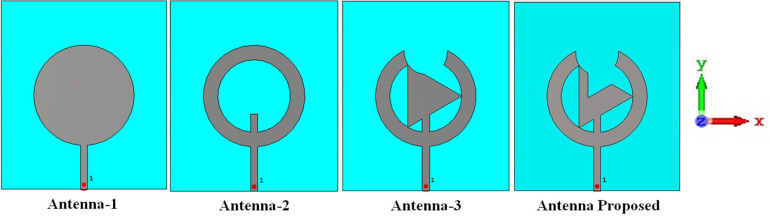
Proposed antenna.

**Fig 2:**
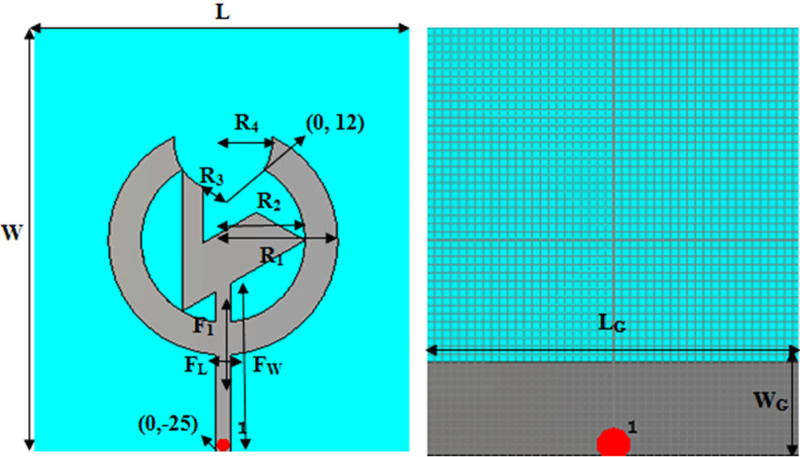
Structure of anticipated antenna.

The triangular slotted antenna circular antenna has the size 52.6 × 46.3 × 1.076 mm^3^ with the 50Ω microstrip line feed. The textile antenna is formed with jeans fabric and used as a substrate to make it more flexible. Further, examine the breast with it for early cancer detection and use it on fabric to monitor the health records of distant patients. The back part of the antenna contains the partial ground plane of 46.3 × 11.3 mm^2^ fabricated from an adhesive copper sheet. An adhesive copper sheet manufactures the flexible antenna's front part on jeans. Fig 1 depicts the steps of forming the presented antenna's design, and Fig 2 illustrates the geometry and background of the proposed flexible antenna.

Refers to Fig 1, which shows the final proposed antenna configuration. Firstly, Antenna-1 is constructed and contains only the circular patch with the feed line. After the next iteration, Antenna-2 is achieved which is the ring-like structure appears as if the circular patch is a slot from the center. Further, join the triangular part (Antenna-3) to the ring and slot the ring from the above section. Finally, slot the triangular structure of antenna three again to obtain the proposed antenna. Fig 2 illustrates the geometrical structure of the patch and the back view of the anticipated antenna with the partial ground, which shows that the length and width of the substrate (jeans) and the ground (copper) are L and W and L_G_ and W_G._ The radius of the outer circle of the patch (copper) is R_1_, and the inner slotted circle is R_2_. The radius of the adjoined triangle is the same as R_2_, and the radius of the circular slot from the ring and the triangular slot from the triangle is R_3_ and R_4_, centered at (0, 12). Moreover, the length and width of the microstrip line feed, centered at (0, −25), are F_L_ and F_W_.

**Fig 3:**
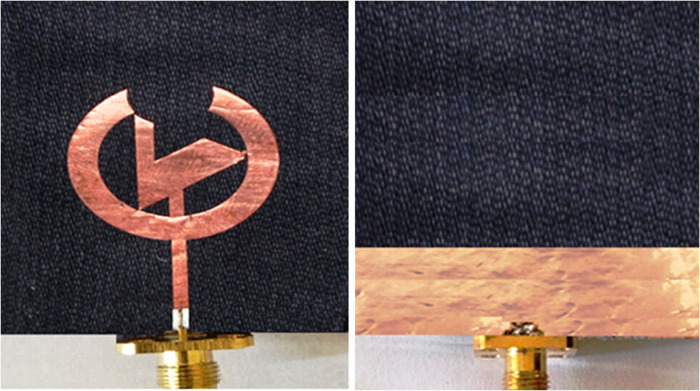
Front and back view of the fabricated antenna.

Here, F_1_ is the length of the minor slot in the feedline, enhancing the anticipated antenna's performance. In addition to the above, Table 2 encapsulates the antenna's geometrical dimensions, and Fig 3 displays the front and back view of the actual model of the proposed fabricated textile antenna.

**Table 2:** Geometrical parameters of the developed antenna.

Parameters	Value
W (Width of Substrate)	52.6 mm
L (Length of Substrate)	46.3 mm
L_G_ (Length of Ground)	46.3 mm
W_G_ (Width of Ground)	11.3 mm
R_1_ (Radius of Outer Circle)	14 mm
R_2_ (Radius of Subtracted Inner Circle)	10 mm
R_3_ (Radius of Triangle Slot)	5 mm
R_4_ (Radius of Circle Slot)	6 mm
F_1_ (Width of Slotted Feed)	13 mm
F_L_ (Length of Feed)	2 mm
F_W_ (Width of Feed)	21.3 mm
H_G_ (Height of Ground)	0.038 mm
h_S_ (Height of Substrate)	1 mm (Jeans)
H_P_ (Height of Patch)	0.038 mm
ℇ_r_ (Permittivity)	1.7
tanδ (Loss Tangent)	0.025

### 3.2 Antenna simulation at different steps

**Fig 4:**
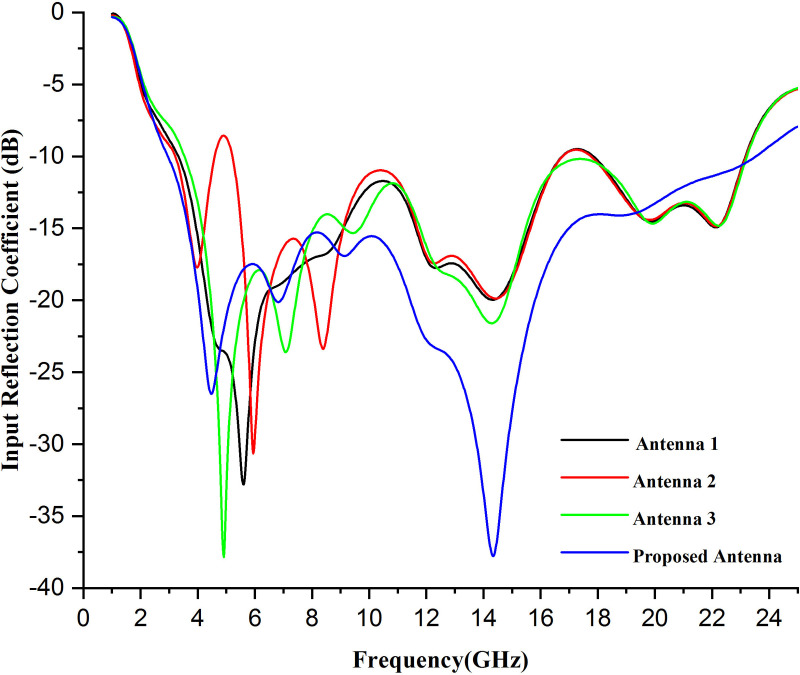
Simulated input reflection coefficient comparison of optimization steps of presented antenna.

Fig 4 displays the performance characteristics and the input reflection coefficient [32] pattern of the presented antenna model at the different frequencies and optimization steps (antenna 1, antenna 2, antenna three, and the proposed antenna). Moreover, Fig 4 implies that antenna 1 in stage 1 has −20 dB of input reflection coefficient at its resonance frequency, i.e., 14.35 GHz. Moreover, antenna 2 in stage 2 has the same input reflection coefficient value at the same resonance frequency. However, the input reflection coefficient value of antenna three increases to −22 dB at its resonance frequency. Finally, the proposed antenna has an excellent value of the input reflection coefficient, i.e., −37.8 dB, and the antenna is flawlessly operated between the frequency range of 2.95GHz to 24.2GHz which shows that the proposed antenna is highly efficient and suitable for different applications which includes fitness tracking (embedded sportswear), healthcare monitoring (tracking vital signs like heart rate, blood pressure, glucose levels), bright clothing (enable communication, navigation connectivity features), telemedicine (remote monitoring, transmitting real-time health data), WBAN's (connecting multiple sensors on the human body and collecting the data for the detection of different health issues, chronic diseases like cancer) and so on. The graph shifts toward the left or backward and becomes smooth at its final stage. (i.e., the proposed stage of the antenna design).

### 3.3 Antenna in the anechoic chamber

**Fig 5:**
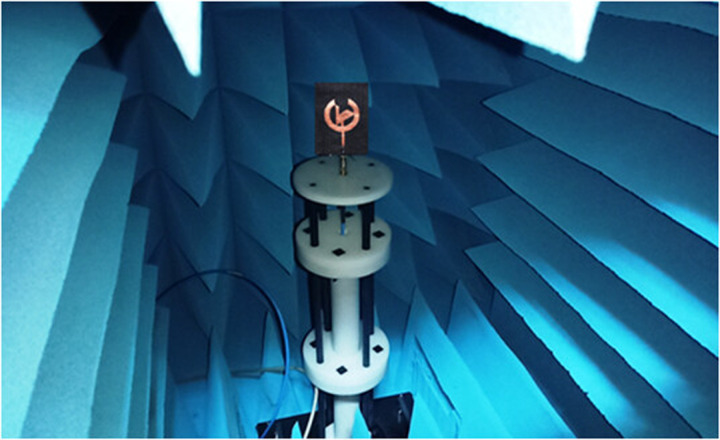
Prepared antenna inside the anechoic chamber.

Wearable antenna testing is conducted in an anechoic chamber, a controlled environment that absorbs electromagnetic waves to eliminate external interference and reflections. This ensures accurate evaluation of parameters like input reflection coefficient, gain, efficiency, and impedance, simulating free-space conditions. The chamber tests the antenna's radiation in various directions, assessing performance across all frequency bands. An input reflection coefficient below −10 dB confirms good impedance matching and minimal signal reflection. Since wearable antennas are closely integrated with the human body, deformation effects and compliance with regulatory standards, such as SAR limits, are also evaluated in the chamber. Together, the anechoic chamber and free-space measurement system are pivotal in enabling wearable antennas to be designed so that they will reliably perform when deployed. Fig 5 displays the presented antenna placed inside the anechoic chamber.

In the section below, we present the evolution of human phantom design models, incorporating flat and breast phantoms, to simulate realistic human tissue interactions and evaluate the antenna's performance in breast cancer detection scenarios.

## 4. Human phantom design models

An advanced human phantom model is a computational or mathematical implementation of the antenna on the body. These models unveil how an occupant interacts with its environment, precisely their radiation pattern against one another. This model is essential for evaluating and optimizing wearable antennas for applications in health monitoring, medical imaging, and communication systems.

### 4.1 Flat phantom model

Human flat phantom models, such as antennas, are simplified body part representations often used in electromagnetic device testing. These models have a flat surface, ideal for simulating interactions between wearable sensors or antennas and the human body. They allow controlled testing of parameters like transmission, reflection, and absorption, particularly in microwave imaging. Constructed with materials mimicking human tissue properties (permittivity and conductivity), these models simulate how radio waves interact with the body. Phantom models are applied in various areas, including antenna optimization for wearable devices like heart rate monitors and glucose sensors and for medical applications like breast cancer detection using microwave imaging. Testing on these models ensures comfort, efficiency, and signal quality (e.g., radiation patterns, SAR) in real-world applications.

**Fig 6:**
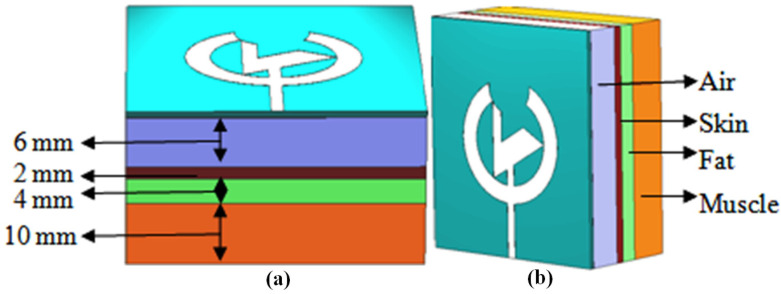
Human phantom model for the presented antenna (a) Top view, (b) Side view.

Fig 6 represents the flat human phantom model of the presented antenna whose dimensions have been considered from the triple band, dual band and mmWave wearable antenna presented by Mitra et al., Ahmad et al. and Khan et al. after experimentation and modification. The model contains the three layers of the human phantom, i.e., skin, fat, and muscle, having sizes 2 mm, 4 mm, and 10 mm. Besides that, when placing the antenna at a distance of 6 mm, the antenna model is simulated using CST software. Note the performance and the SAR value, which is crucial in developing and testing wearable antennas. This SAR ensures that these devices function effectively and safely when used in real-world scenarios on the human body. Table 3 outlines the specifications of the flat phantom model.

**Table 3:** Specifications of the flat human phantom model.

Tissue	Skin	Fat	Muscle
Permittivity (ℇ_r_)	42	5.2853	52.791
Conductivity (S/m)	1.5618	0.10235	1.705
Loss Tangent	0.2725	0.1450	0.24191
Density (kg/m^3^)	1109	911	1090
Thickness (mm)	2	4	10

### 4.2 Breast phantom model

**Fig 7:**
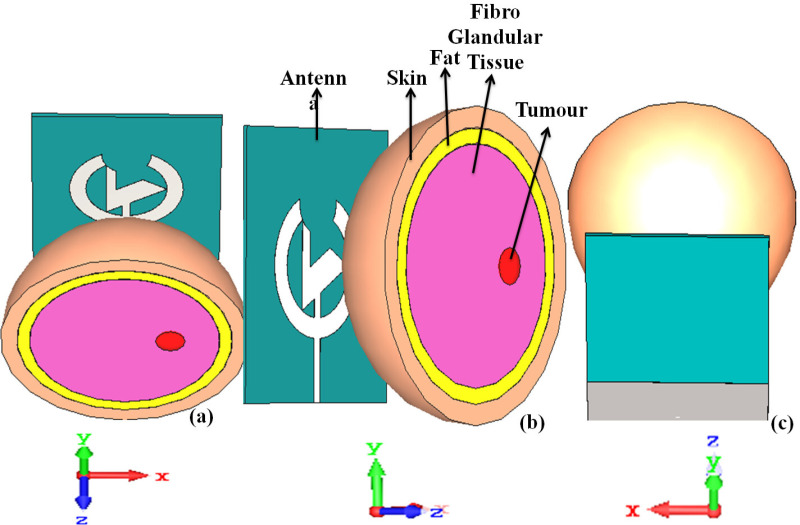
Breast phantom model with tumor tested with the presented antenna.

The breast phantom model is designed to simulate the anatomy and electromagnetic properties of the breast, making it ideal for developing and evaluating new medical imaging techniques. Breast cancer remains a leading cause of death among women globally, with cancerous cells spreading to nearby tissues and lymph nodes, eventually to other body parts, such as the lungs, bones, and brain, particularly in advanced stages (stage 4). Early diagnosis is critical for improving cancer outcomes, and breast phantom models are used to safely assess and optimize antenna performance for wearable detection devices. These systems ensure that outcomes are practical, accurate, and non-hazardous for patients [30]. Fig 7 exhibits the breast phantom model with a tumor and its simulation with the antenna presented on CST software. The phantom out of commodities chemically similar to the dielectric properties of tissue and imitation targets as tumors. Breast phantoms are the simplified anatomical structures of the human body and can incorporate skin, fats, and glandular layers. They can also include non-invasive tumors or some other pressing factor. Model complexity may range from homogeneous (uniform material combination) to more heterogeneous phantoms that capture the entire progression of breast morphology.

On top of that, phantoms are essential for developing wearable breast cancer detection systems as they can be used to test how well the antennas detect and localize tumors, verifying that not only is it able to find small targets efficiently but also remains harmless by SAR calculation. Table 4 illustrates the required specifications for preparing the breast phantom model. Creating phantoms enables imaging algorithms, antenna design, and system performance to be optimized before clinical trials or deployment in real-world environments. Researchers have previously tested microwave-based breast cancer detection techniques using phantoms to determine the effectiveness of the systems in detecting tumors of various sizes and depths.

**Table 4:** Specifications of breast phantom model.

Tissues	Permittivity (ℇ_r_)	Electrical Conductance (S/m)	Density (ρ) Kg/m^3^
Skin	36.7	2.34	1109
Fat	4.84	0.262	911
Fibro glandular	11.5	0.5	1050
Tumour	54.9	4	1058

This section presents the parametric analysis of the proposed antenna, exploring the impact of key design parameters on its performance to ensure optimal functionality for wearable applications.

## 5. Parametric analysis of the proposed antenna

Researchers systematically explore parameterized studies, showing that variations in parasitic loops help to understand antenna performance. Such a study is necessary to tune the antenna's properties to meet the performance standards and requirements, especially well-being monitoring and communication on top of an array of medical imaging and cancer detection. Researchers alter the physical dimensions of the antenna to investigate their role in resonance frequency, bandwidth, and radiation pattern. The antenna performance is enhanced for a particular application by surveying various shapes, such as rectangles, circles, and ellipses. Therefore, one meaningful way to analyze wearable antennas is through parameterized study. A parameterized study of a particular antenna helps adjust the changeable parameters to universally perform across conditions and applications with the best results from the designed point of view. Significant parameters can be systematically and systematically analyzed to design such antennas for wearable technology safely. Parametric analysis modulates an antenna's specific absorption rate (SAR) so that the changes in various parameters like substrate material, geometry, feed position, and frequency affect a change in electromagnetic field deposition pattern and magnitude. The SAR is directly associated with these changes. Adapting these will help reduce SAR levels, which could be safely used in wearable antennas while maintaining good performance.

### 5.1 Variation in feed length

The feed length variation of a wearable antenna greatly influences impedance matching, resonance frequency bandwidth, and radiation pattern. Altering the feed length may make the impedance match better or worse; this affects how well the return ratio can be shown from the antenna end to the system side. Moreover, the longer feed can help lower the resonance frequency, while a shorter one will increase it. A feed of proper length can increase the bandwidth and ensure that more frequency bands are effectively radiated from this antenna. On the other hand, an improper feed length can decrease bandwidth, changing the current distribution on top of the antenna and might result in a difference in the radiation pattern. If the feed length is not well matched, higher SAR may be generated; therefore, it is essential to select the best match so that both performance and safety can run in unison.

**Fig 8:**
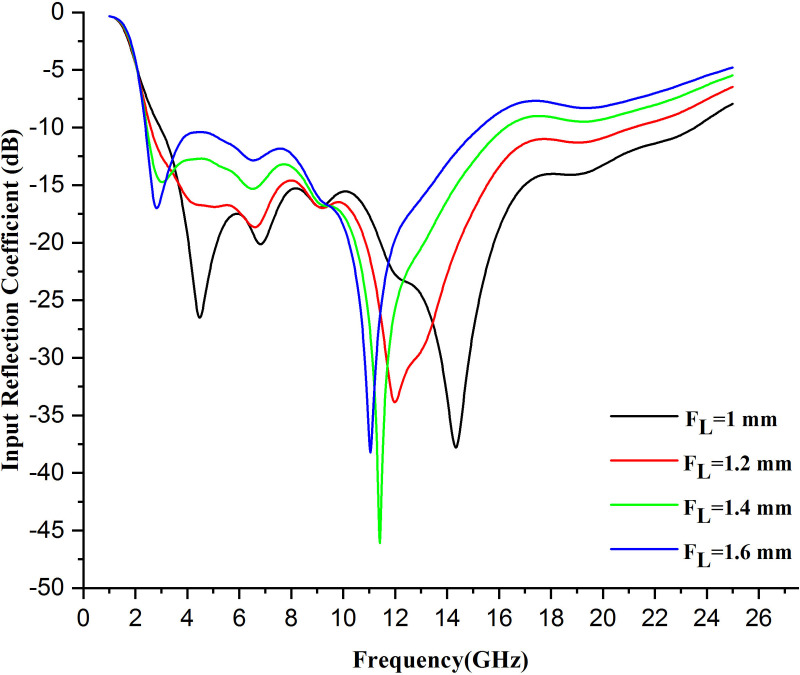
Variation in input reflection coefficient on changing length of feed by .2 mm.

In the proposed work, on increasing the feed length by .2 mm, the input reflection coefficient performance of the antenna is affected, and the graph is shifted upward when FL =  1.2 mm. When FL = 1.4 mm, the antenna reflects a reasonable input reflection coefficient performance, i.e., −45 dB at 11.2 GHz. Further, at FL =  1.6 mm, the graph is again shifted backward at the same input reflection coefficient level at the frequency of 10.8 GHz. This shift is because of the resonance frequency shift, which lengthens the feed and increases the electrical path for the signal to travel. Longer path lengths may decrease the resonance frequency of the antenna, which will shift the peak to lower frequencies. If we increase the feed length, the graph shifts the element to the left. Moreover, impedance mismatch, phase delay, and coupling with a ground plane or substrate can also result in the backward and up shift of the graph. Fig 8 mentions the shift in the graph on increasing the length of the feed on an antenna.

### 5.2 Variation in width of the ground

**Fig 9:**
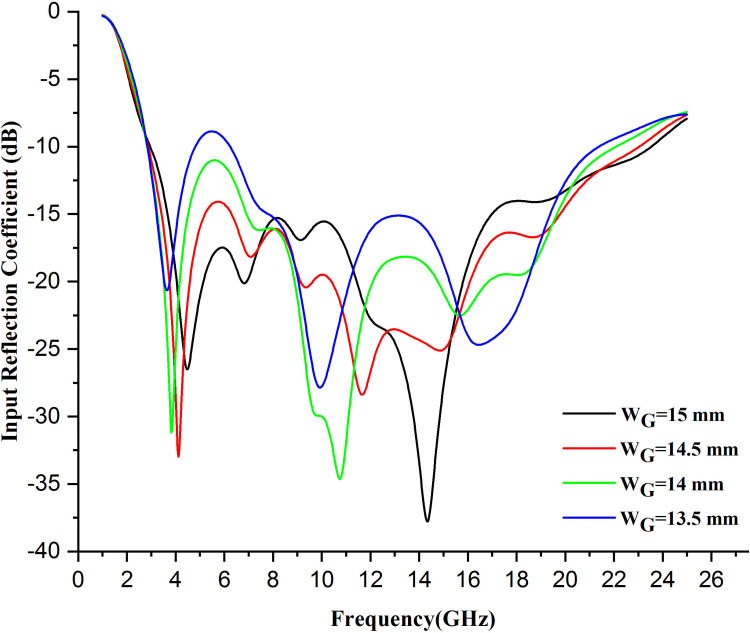
Variation in input reflection coefficient on changing width of ground by .5 mm.

Changing the width of the ground plane in a wearable antenna can have several effects on its performance. Moreover, separating the ground plane width in wearable antennas is a crucial design feature. Typically, widening the ground reduces resonance frequency, which results in a narrow bandwidth, making the radiation pattern more directional. Conversely, decreasing the width can increase resonance frequency and broaden bandwidth while the radiation pattern becomes more approximated to the omnidirectional pattern. The ground plane width also affects the efficiency and SAR values, as both needs to be balanced carefully for optimum antenna performance. Fig 9 shows the distortions in the graph when changing the width of the ground of an antenna.

Here, when the width of the ground decreases, the graph goes up and down and shifts towards the left and sometimes upwards, increasing the resonant frequency and bandwidth of the antenna. Additionally, this will improve impedance matching, depending on the specific design. However, it may also decrease efficiency and increase SAR values, which are essential factors to consider in wearable antenna applications.

### 5.3 Changing the radius of the outer circle

Changing the radius of the outer circle, such as a circular patch element, can significantly affect several performance parameters associated with an antenna. Change in the outer circle of an antenna mainly affects its resonant frequency (wavelength) bandwidth, radiation pattern, and gain. A higher radius generally leads to lower resonant frequency and more considerable gain, and a narrower beam width lowers the radius and has the opposite effect. It needs to be carefully optimized, especially for applications where the size and SAR are essential.

**Fig 10:**
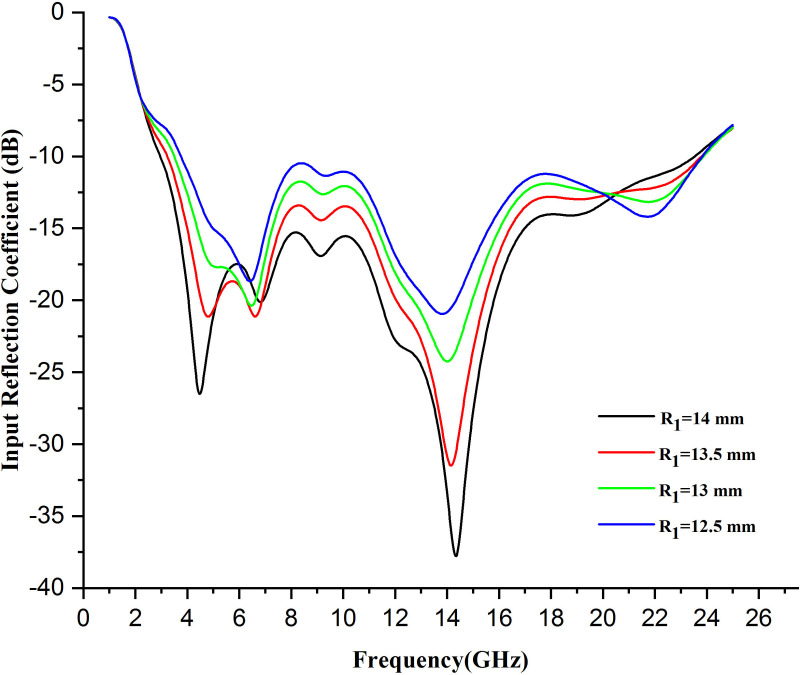
Variation in input reflection coefficient on changing radius of outer circle R1 by 0.5 mm.

The plot illustrations shown in Fig 10 manifest that when the radius of the patch of the proposed antenna is decreased; it results in an upward shift of the antenna. Generally, when the patch radius becomes smaller, this also illustrates an upward shift in the graph of a wearable antenna and suggests poor impedance matching causes higher resonant frequency and narrowing bandwidth that results in much more surface wave losses, thereby causing less radiation efficiency but surging SAR. This results in less effective performance, as demonstrated by the trajectory up and to the right.

This section presents and analyzes the experimental results obtained from the wearable antenna designs, highlighting the key findings and their implications for future applications in wireless communication and medical fields. Detailed discussions are provided on the performance metrics and potential improvements.

## 6. Results interpretation and discussion

### 6.1 Input reflection coefficient of the presented antenna

The input reflection coefficient behavior of a wearable antenna directly affects its performance and efficient operation. Typically, input reflection coefficient of −10 dB or better over the desired frequency bands ensures minimal reflections and power radiates most effectively. Good design, proper material selection, and environmental consideration are vital in obtaining wearable antennas with excellent input reflection coefficient performance. The wearable textile antenna's standard input reflection coefficient plot shows how well it matches the transmission line over its working frequency range. The x-axis of the plot displays the band at which the antenna is used to operate, generally in GHz or MHz. The y-axis represents the input reflection coefficient in dB. Small negative values are used in plotting [31,32].

Moreover, the antenna's performance with power transfer is better when input reflection coefficient becomes more negative. The points where the input reflection coefficient drops substantially below −10 dB are the frequencies at which that antenna is well-matched. The frequency bandwidth in which the input reflection coefficient is still smaller than −10 dB is the frequency range that exhibits the antenna's effective area.

**Fig 11:**
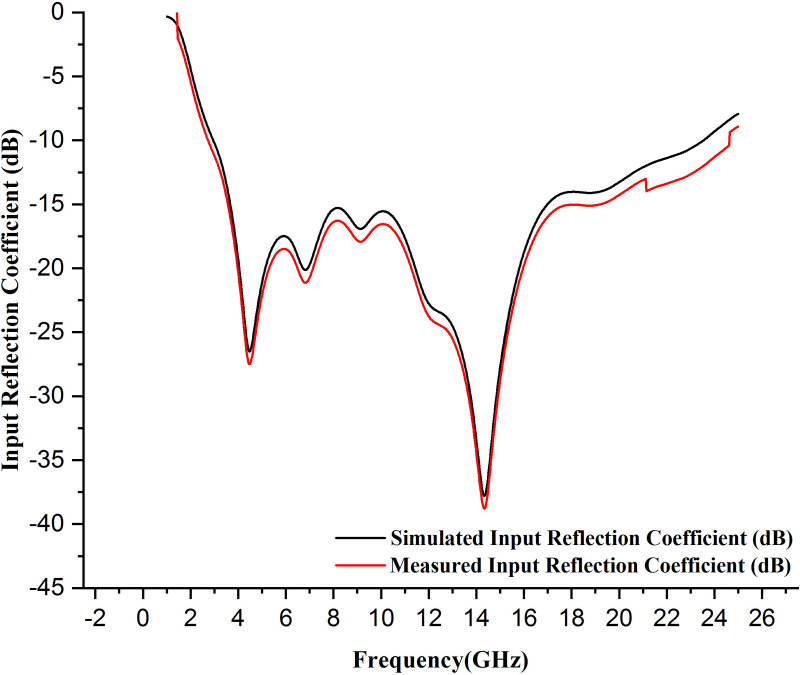
Measured and simulated input reflection coefficient of the presented antenna.

**Fig 12:**
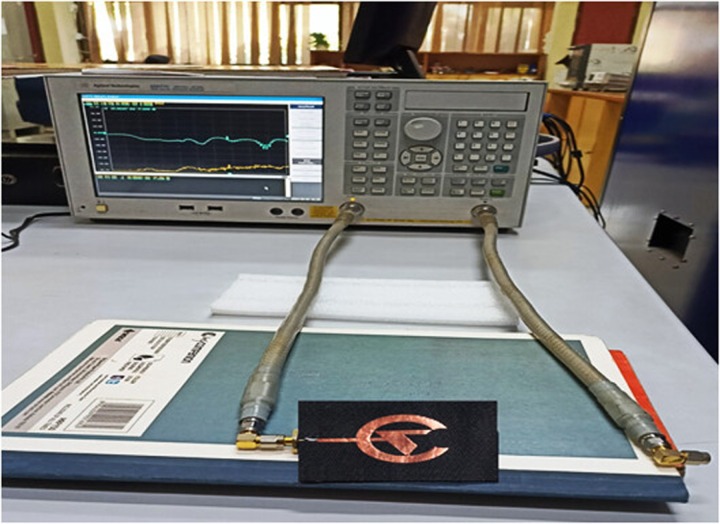
Input reflection coefficient measurement of the prepared antenna on VNA.

Here, the present antenna's input reflection coefficient is obtained by simulating the prepared design on the CST software. Secondly, real-time analysis and measurement can be done by connecting the antenna with the vector network analyzer (VNA) and taking the measurements using the software. The simulated and measured plot is disposed of in Fig 11, and real-time VNA measurements are depicted in Fig 12, which illustrates that the antenna resonates between the wide frequency range below −10 dB and the resonance frequency of the antenna, is 14.35 GHz with a maximum −37.8 dB input reflection coefficient performance. Then, the antenna's bandwidth is 66.88%, which manifests that the antenna is suitable for cancer detection and WBANs, biotelemetry, and healthcare applications. Moreover, the measured and simulated input reflection coefficient is similar, meaning the simulated antenna is perfect for real-time analysis.

Although the close alignment of simulated and measured results in Fig 11 accentuates the robustness of the propounded antenna design. This remarkable agreement, while unprecedented, stems from precise fabrication techniques, rigorous VNA calibration, and explicit simulation modeling that mirrored experimental conditions. Minor deviations were minimized through meticulous design and measurement processes.

### 6.2 Current distribution in the presented antenna

The surface current distribution on a wearable antenna is essential to predict its radiation performance and how it may be affected by materials, bending, and presence near the human body. Additionally, current distribution illustrates how the electric current moves along the radiating elements of an antenna through its surface. The substrate material (e.g., a fabric cotton, polyester, or jeans) and the conductive materials employed within them (i.e., copper /conductive threads) play a role in how surface currents are distributed through fabrics. Changes in the dielectric constant of the substrate material can alter current distribution, radiation pattern, and antenna efficiency. The antenna's resonance frequency and radiation characteristics can be shifted, potentially by bending its current paths, which changes the effective length of the current path. Near-field surface currents depend on tissues ' dielectric properties when the antenna is attached to the human body. This proximity can give the detuning of antenna configurations that will degrade its input reflection coefficient, gain, and efficiency. The surface current distribution is generally exposed to electromagnetic simulation tools, such as CST microwave studio and high-frequency simulation software (HFSS), that can show the amount of curl on a contour layer throughout the antenna. Additionally, measurements can be taken with special devices to verify the simulation results.

**Fig 13:**
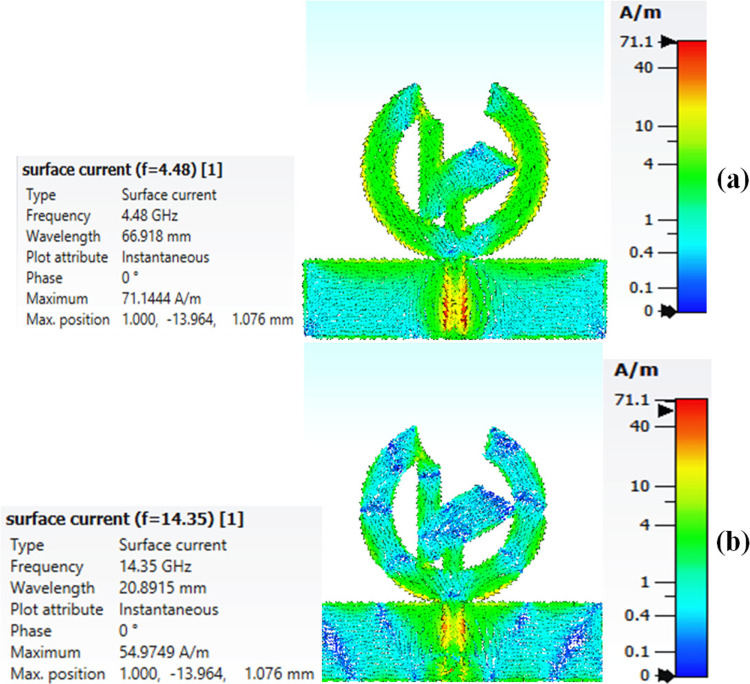
Current distribution of the presented antenna at its resonance frequencies (a) 4.48 GHz and (b) 14.35 GHz.

Fig 13(a) and b illustrates the current distribution across the proposed antenna at the two frequencies 4.48 GHz and 14.35 GHz, which represents that the amplitude of current across the feedline is about 71.1 A/m at the frequencies 4.48 GHz and 14.35 GHz and the current ranges from 40 A/m to 0 A/m is distributed evenly across the antenna at both the frequencies determining the antenna's good performance and reliability, especially under varying conditions encountered in practical use.

### 6.3 Measured and simulated radiation pattern of the proposed antenna

The radiation pattern of a wearable textile antenna is an essential feature for its practical operation since it shows how the electromagnetic energy emitted by the source has been radiated in different directions. This type of antenna integrated into clothes or fabrics is usually more comfortable and even available for visitors simultaneously. Most wearable textile antennas have a directional or semi-directional radiation pattern, thus directing energy in specific directions. It helps optimize signal strength, which reduces interference when the antenna is body-worn. Moreover, the radiation pattern is a function of the distance between the antenna and the human body. The human body's dielectric properties can also absorb and reflect electromagnetic (E.M.) waves, which may induce deformation in pattern shape, potentially decreasing radiation efficiency. Also, developing wearable textile antennas requires considering the influence of surrounding materials, such as clothing, in daily life and orientation when placed on or near a human body, and this aims to obtain the radiation pattern that satisfies the application's needs for communication, health monitoring, or any other use. Thus, the antenna's design, material properties, and interaction with the human body influence the radiation pattern of wearable textile antennas. Achieving a reliable and efficient radiation pattern in these flexible, body-worn devices requires careful consideration of these factors to ensure optimal performance in real-world conditions.

**Fig 14:**
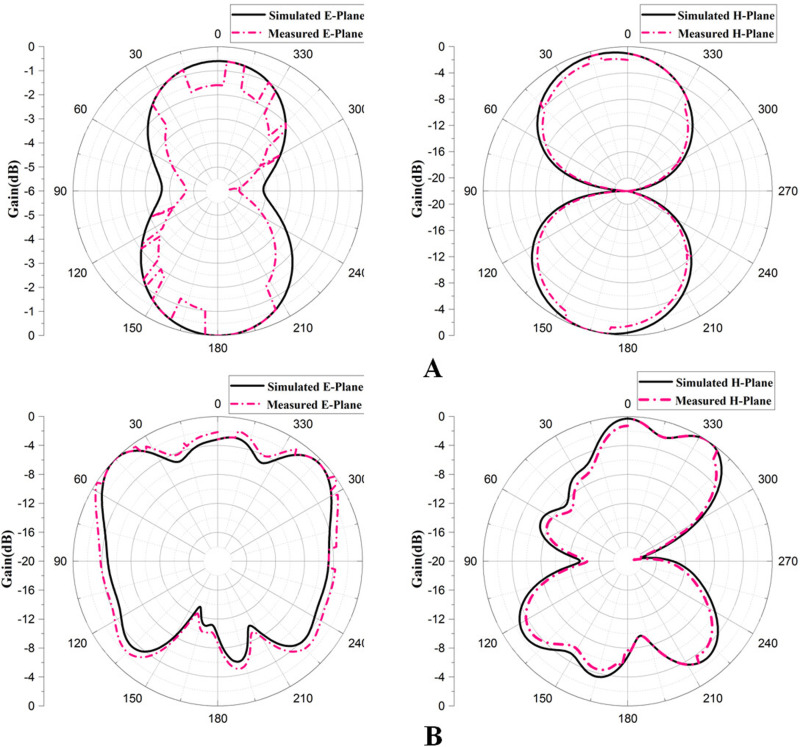
Simulated and measured radiation pattern of the presented antenna at (A) 4.48 GHz and (B) 14.35 GHz.

Fig 14 displays the antennas simulated and measured polar E-plane and H-plane radiation patterns at 4.48 GHz and 14.35 GHz resonant frequencies. Additionally, Fig 14 demonstrates that at the lower frequency, 4.48 GHz, the antenna shows the directional radiation pattern, but at the higher frequency, 14.35 GHz, the antenna exhibits the omnidirectional radiation pattern with main and back lobes, and the antenna resonates in all the directions. Omnidirectional antennas provide uniform coverage in all directions, making them suitable for general-purpose communication. In contrast, directional antennas focus energy in a specific direction, offering more excellent range and reduced interference, which is ideal for targeted communication needs. Furthermore, the presented polar plot of an antenna mentions that the simulated E and H- planes of the antenna at both frequencies are somewhere similar to the measured radiation plot of the presented antenna, which means that the simulations in the CST environment are similar to the measured real-time radiation plot measured on VNA.

### 6.4 Gain, directivity, and radiation efficiency of the presented antenna

Parameters like gain, directivity, and radiation efficiency are significant in the performance characteristics of any antenna, whether it be a semicircular slotted wearable textile (SWT) or another instance. Gain measures the carving ability of an antenna to concentrate input power for generating radio waves in a particular direction. Decibels (dB) typically measure gain. The higher gain causes more input power in a specific direction, which is significant for wearable antennas. Their use involves body effects or presence near radiating materials, causing the signal strength at various regions around them to be disrupted frequently due to continuous movement. The gain (G) can be measured as the ratio of radiation efficiency (η) to the directivity (D), as shown in Equation 9.


G=ηD (9)

Besides that, directivity quantifies the extent to which an antenna's radiation pattern is preferentially in one direction. It is the degree of the radiation intensity in one direction to a reference point, comparing across all other directions. It is an essential parameter for some applications of interest. It can be efficiently employed to determine how well the wearable textile antenna radiates or receives signals from a given direction, as this happens naturally with bright e-textile clothing.

**Fig 15:**
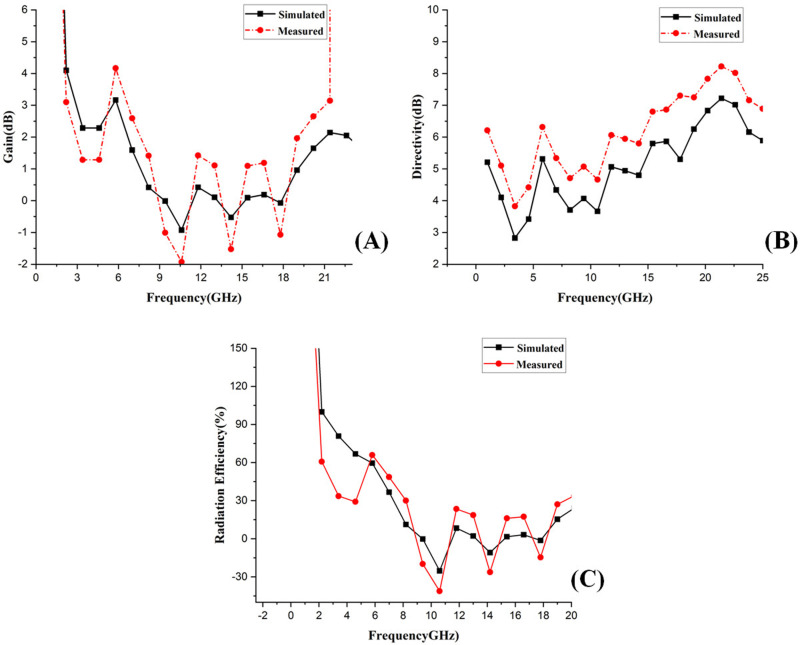
Simulated and measured (A) Gain, (B) Directivity and (C) Radiation efficiency of the presented antenna at different frequencies.

Moreover, the radiation efficiency is the ratio of the power that comes out from an antenna to send some signal and the total input power of the antenna, and it includes losses due to materials (the textile substrate) and the antenna's design parameters. Some materials employed in wearable antennas have higher losses than those used for conventional antennas. In wearable applications, this is critical as high radiation efficiency implies that the antenna effectively radiates most of the power it receives, facilitating a good performance. Fig 15(a), 15(b) and 15(c) depict the simulated and measured gain, directivity, and radiation efficiency plot which shows that the presented antenna has a peak gain of 3.16 dB at 5.8 GHz with the peak directivity of 6.5 dB and 8 dB at 5.8 GHz and 24 GHz and radiation efficiency of more than 150% at 2.5 GHz, 59.56% at 5.8 GHz and 66.88% at 4.48 GHz respectively. Likewise, the plot of the gain, directivity, and radiation efficiency can exhibit fluctuations (going up and down) due to the complex interplay between the antenna's design, the materials used, the environment in which the antenna operates, and the frequency-dependent behaviors. Understanding and mitigating these factors is crucial for optimizing the antenna's performance. In Wearable antennas, particularly those with complex shapes such as slots, patches, or other geometries, multiple resonant modes can be excited simultaneously. Due to this, the radiation patterns of each mode will have a different shape, and hence, directivity may change at various frequencies as we excite these modes. Furthermore, the resonant modes of the antenna change as a function of frequency and are responsible for how well an antenna focuses energy in different directions. In addition, changes in the dielectric constant, physical deformations, body proximity effects, interaction with nearby objects, and narrow bandwidth for several frequencies can influence the antenna's effective radiation pattern and lead to fluctuation in a directivity plot. Table 5 represents the simulated gain, directivity, and radiation efficiency values.

**Table 5:** Simulated values of gain, directivity and radiation efficiency of the proposed antenna.

Frequency (GHz)	Gain (dB)	Directivity (dB)	Radiation Efficiency (%)
2.5	3.05	4	150
5.8	3.16	6.5	59.56
4.48	1.5	3.5	66.88
14.35	1.25	5	20

Besides that, the plots of gain, directivity, and radiation efficiency are indistinguishable in all ways; only there are specific peaks mismatch, which does not cause any change in the results and applications, and the presented antenna is perfect for the applications of cancer detection, telemedicine, remote health care monitoring and for all body-centric wireless communication (BCWC).

### 6.5 Bending analysis of the presented antenna

Bending analysis of wearable antennas is essential due to their deformation effect on the human body. Bending can significantly impact wireless properties, affecting resonance frequency, input reflection coefficient, radiation pattern gain, and specific absorption rate (SAR). Placing the wearable antenna over curved human tissues distorts the antennas’ physical size and radiation lengths upstream. That can cause a change in the resonant frequency. Bending may cause a change in input reflection coefficient, probably due to the excitation mismatch between the antenna and transmission line or feeding network, forcing more reflections and lower power transfer efficiency, resulting in worse input reflection coefficient. Due to bending, poor impedance matching leads to low performances and often results in decreased gain due to the variations of effective aperture and radiation pattern. Further, bending can alter how the antenna interacts with the body and, in some cases, increase SAR at specific regions. Also, this is important for how much R.F. energy a body absorbs, and the safe limit should not be exceeded. By characterizing how bending impacts the antenna, engineers can ensure that their designs will be robust and reliable even when worn on a human body. With the mix of materials and careful optimization of antenna geometry, it is possible to mitigate this performance degradation due to bending and maintain impressive real-world (3D) radiated efficiency. In the presented antenna, both sides of the cylindrical bending of the antenna are performed in CST software at the radius of 4 cm, i.e., 40 mm, and we are obtaining an excellent input reflection coefficient performance of the presented antenna.

**Fig 16:**
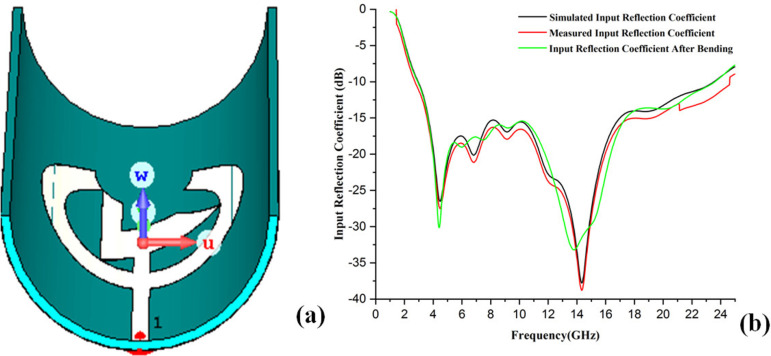
(a) Bending analysis and (b) Input reflection coefficient performance after bending of the anticipated antenna.

Fig 16 shows the bending done at 40 mm in the CST environment and the proposed antenna's simulated, measured, and bending input reflection coefficient performance. The presented antenna is simulated again in the CST environment, and the input reflection coefficient parameters obtained are more than satisfactory; this confirms that the presented antenna is bent correctly on the human arm or breast or the cloth, and thus, it is further helpful for the breast cancer detection as well as healthcare monitoring and WBAN's. Thus the above bending analysis confirms that the wearable antenna presented in this research is designed as a proof of concept for breast cancer detection using UWB technology. While the results provided in the paper are based on modeling and simulations, they are grounded in established electromagnetic principles and real-world material properties. The authenticity of the antenna's performance is demonstrated through simulations and analysis, which are indicative of its potential for practical application. However, further experimental validation, including clinical trials, will be necessary to confirm its real-world effectiveness and accuracy for healthcare monitoring.

### 6.6 SAR analysis

The specific absorption rate (SAR) in the case of wearable antennas is the amount of radio frequency (R.F.) electromagnetic energy a human absorbs when exposed to R.F. radiation from an antenna. It is the benchmark for examining how much E.M. waves are absorbed by tissues. SAR is the standard level to measure how many electromagnetic waves can be packed into human tissues when exposed near an antenna. Normal restrictions have been recognized to point out the secure ranges of SAR. SAR levels were judged based on USA and European standards. For wearable antennas, the design must be in such a way that its SAR will remain below the standards of the USA established by the Federal Communication Commission (FCC), i.e., 1.6 W/Kg and the EUROPE standard established by the International Commission for Non-ionizing Radiation Protection (ICNIPR), i.e., 2 W/kg for optimal designing purposes [32]. The human phantom model design mimics the characteristics of a human body during simulations. Also, the closer to our body that we hold an antenna, the higher the SAR radiation levels will be fixed. Antennas must limit the amount of energy they absorb while maintaining performance. The conductive and dielectric properties of materials used in wearable antennas affect SAR values. Furthermore, SAR generally increases with frequency, operating mode, and top transmission powers. Wearable antennas will be subject to bending and deformation, further changing their electromagnetic performance, hence changing the SAR. Thus, SAR estimation provides a safe exposure level to electromagnetic radiation and ensures that it will not deteriorate the wireless communication of wearable antennas.

#### 6.6.1 SAR analysis on flat human phantom model.

Flat phantom models allow the SAR to be tested for wearable antennas in a repeatable way, guaranteeing that such devices can be operated safely close to the human body.

**Fig 17:**
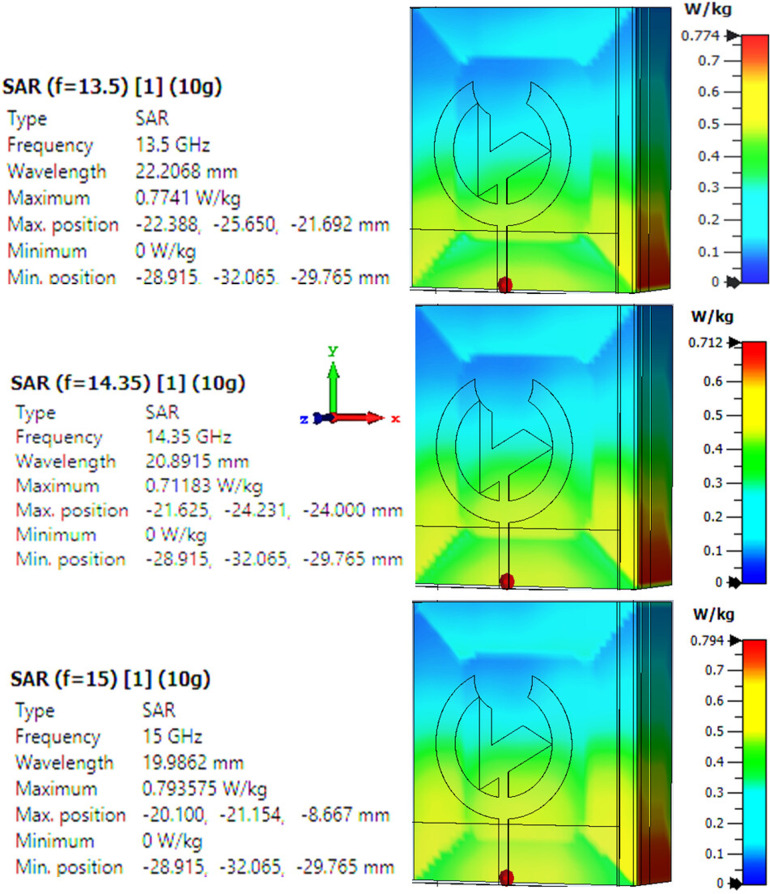
SAR value for the presented antenna at different frequencies in the flat phantom model.

The flat phantom model is designed to measure the SAR of a source in terms of safety for wearability antennas. The flat phantom model simulates human Tissue to determine how much electromagnetic energy gets absorbed when it is on or near the body. It is important because it matters whether the total power of all wearable devices, once used, adds up to an amount that exceeds the regulatory limits for electromagnetic exposure. Fig 17 exhibits the SAR calculations at the two distinct frequencies and one resonance frequency, simulated in the CST environment. Here, the best acceptable SAR value at the resonance frequency of the antenna, i.e., 14.35 GHz, is 0.712 W/Kg for 10 gm of Tissue. The rest of the SAR values are 0.774 W/Kg and 0.794 W/Kg for 10 gm of Tissue at the frequencies 13.5 GHz and 15 GHz, which also seem to be the satisfactory values, and all these values fulfil the required estimated values for the desired WBANs and healthcare applications as they are under the safety limits.

#### 6.6.2 SAR analysis on breast phantom model.

The specific absorption rate (SAR) evaluation of wearable antennas for breast health monitoring and other biomedical applications is a crucial process, which generally requires performance measurement inside an anatomically realistic that mimics the human body. The breast phantom model is designed to simulate the dielectric properties of human breast tissue and can be used to evaluate R.F. power absorbed while operating an antenna near breasts.

**Fig 18:**
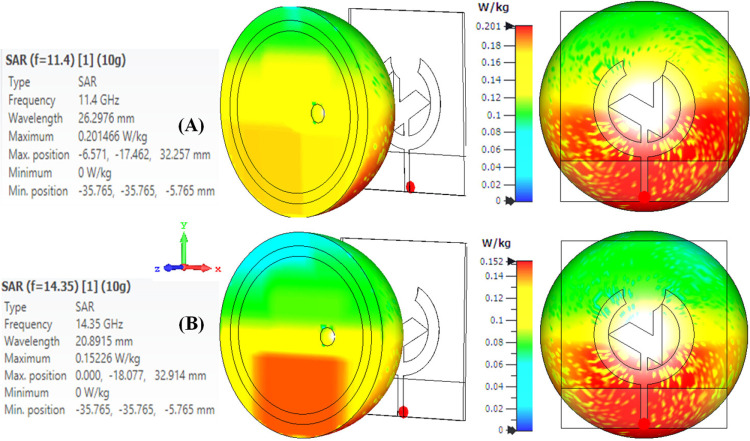
SAR value of the presented antenna when applied on the breast phantom model at (A) 11.4 GHz, (B) 14.35 GHz.

Fig 18(a) and (b) represent the SAR value at two frequencies: its resonance frequency and the requisite SAR value obtained under the secure limits. The proposed antenna exhibits a 0.201 W/Kg SAR value at the frequency of 11.4 GHz and 0.152 W/Kg at the resonance frequency of 14.35 GHz, which is perfectly under the USA and EUROPE standards. Thus, this is a breast phantom SAR study to ensure the subsequent wearable antennas for breast health monitoring are safe and compliant with acceptable E.M. exposure. Antenna design, frequency of operation, and distance from the target are essential in lowering SAR to be satisfactory to the affected person's protection.

#### 6.6.3 Radiation pattern of breast phantom model.

In simulating the radiation pattern of a wearable antenna on a breast phantom model, several factors affect how much energy the wireless appliance radiates through tissue. Any breast phantom model, posing the same characteristics as human tissue material near an antenna, can influence radiation patterns greatly due to its dielectric or absorption properties.

**Fig 19:**
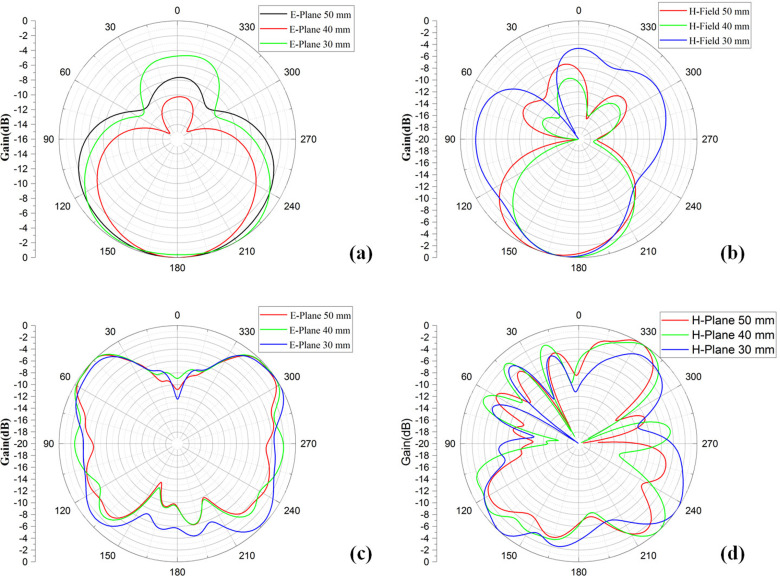
Simulated radiation pattern of the presented antenna at (a), (b) 4.48 GHz and (c), (d) 14.35 GHz when it is applied on breast phantom model.

Fig 19 illustrates the simulated E-plane and H-plane polar radiation pattern, taken by placing the proposed antenna at three different places from the breast phantom model, i.e., at 50 mm, 40 mm, and 30 mm. Observing an isotropic, directional, and omnidirectional radiation pattern at 4.48 GHz, and isotropic and directional lobes attain 14.35 GHz. Thus, the radiation pattern of a conventional wearable antenna on the phantom always differs from that in free space.

The shift from free space to the actual body will cause several practical problems, such as reduced gain, asymmetric radiation due to energy absorption by tissue, and shifted directivity. Accurate modeling of these effects is essential, simulating the antenna performance for human health monitoring on wearable applications such as breast health monitors.

#### 6.6.4 Input reflection coefficient performance of the antenna by placing it at different distances from the breast.

When the wearable antenna is placed at varied distances from a breast phantom, the input reflection coefficient response varies due to impedance matching and coupling between the antenna and phantoms.

***Close proximity to the human body:*** Closer to the breast phantom, electromagnetic waves interact more with the tissue, which causes variations in the input impedance of this antenna. Moreover, for smaller distances, the absorption of electromagnetic waves by the breast tissue is increased, which results in higher input reflection coefficient (a lower dB value). Also, the tissue would shift the antenna's resonant frequency, causing its designed operating frequency to no longer align perfectly. Moreover, if the phantom is too close to the antenna, there will be too much coupling, causing unwanted signal reflection and degrading the input reflection coefficient.

**Fig 20:**
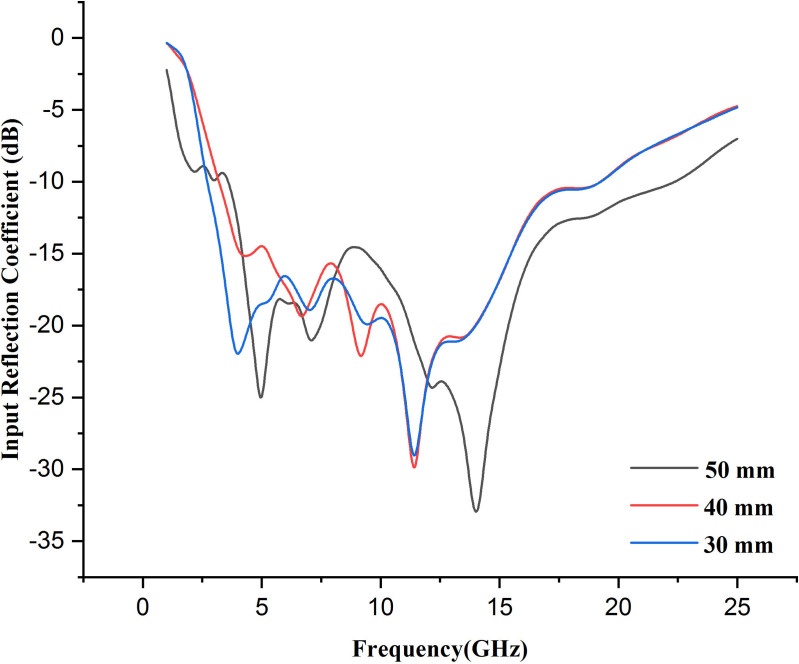
Input reflection coefficient comparison on keeping the presented antenna at different position from the breast phantom.

***Farther from the human body:*** The reduction in coupling between the antenna and breast tissue translates to less loss and lower reflections, which improves input reflection coefficient at longer distances. Removing the antenna slightly from the phantom leads to a better match between an antenna and its feed line, improving input reflection coefficient performance. In addition, when the distance increases, the tissue interacts less with energy, and input reflection coefficient improves because less radiation is absorbed. However, separations that are too high can result in lower performance detection or monitoring. Fig 20 presents the plot of input reflection coefficient performance on placing the antenna at discrete places from the breast phantom model. The input reflection coefficient of the antenna changes on changing the distance. Also, as the distance decreases, the resonance frequency shifts towards the backward direction by changing the antenna range to 11 GHz with −29 dB return loss.

### 6.7 Discussions

Designing and simulating the triangular-slotted circular UWB flexible antenna for breast cancer detection and healthcare applications shows advanced progress in wearable antenna technology. The developed antenna has demonstrated that it can offer a wide band around 2.95–24.2 GHz with a bandwidth of approximately equal to156% and shows good potential for medical applications. Its 46.3 × 52.6 × 1.076 mm^3^ size and bending properties make it appropriate for healthcare monitoring and breast cancer detection. If the antenna lacks a wide range of frequencies and is more prominent in size, this would reduce the quality of high-resolution images. Also, the antenna will be restricted to specific communication and protocols as the devices cannot support the multiple band frequency.

Moreover, an antenna that is too large or cumbersome to wear would be awkward. The user would need to stay longer for it to function as a helpful tool. Such a design would also restrict its usability for permanent health monitoring or fitness tracking since it may be uncomfortable to wear. Also, the large size results in performance degradation and high-power consumption; therefore, the presented antenna is compact, resulting in many advantages. The most significant requirement for these antennas is that they should be small enough to be hidden within clothing or wearable devices. An antenna that is too big may be too stiff for use in intelligent fabrics or wearable medical technology.

The antenna exhibits input reflection coefficient of −37.8 dB at its resonance frequency of 14.35 GHz. It indicates the efficient radiation properties that improve transmission with minimum signal reflection or impendence matching, making it suitable for its potential applications. Therefore, the antenna is desirable for microwave imaging and cancer detection. Bending tests have shown that the antenna retains its performance after deformation. The antenna's flexibility ensures it can instantly conform to the body by delivering reliable performance and additional validations required for real-world healthcare scenarios. This adaptability offers comfort for its wearers but maintains functional integrity simultaneously. In addition, the SAR values of 0.712 W/kg for the flat phantom model and 0.152 W/kg breast phantom models are significantly below the safety limits. This value of SAR confirms that the antenna is also safe under prolonged use at a near distance from human tissue in daily life operations. The small SAR values demonstrate for the first time in detail how effectively a wearable medical device can minimize electromagnetic exposure. In addition, if the SAR value exceeds 2 W/Kg, it can cause immoderate heating of the body tissues, leading to thermal damage, discomfort, and burns. Long-term exposure to high SAR values can also affect the nervous system and other biological effects. Therefore, keeping SAR levels within safe limits is crucial for wearable antenna design and usage. Alternatively, the microwave imaging application shows the potential of using this antenna as an inexpensive and non-invasive substitute to regular screening methods for the detection of breast cancer. Miniaturization, comprehensive frequency coverage, and flexibility of the presented antenna will pave the way for future wearable healthcare devices that can act as an excellent tool in continuous women's healthcare monitoring, including early breast cancer detection.

### 6.8 Future plans

Future plans for this research include testing the proposed triangular-slotted UWB flexible antenna design on human subjects to validate its effectiveness in breast cancer detection. The results from these tests will be compared with traditional mammography images to assess the accuracy and reliability of the antenna. This comparison aims to establish the antenna as a non-invasive, radiation-free alternative or complement to mammography. Additionally, efforts will focus on optimizing the design for real-world applications, ensuring it meets clinical standards and is suitable for widespread healthcare use. These steps will help transition the technology from simulation to practical implementation.

This section presents a comparative analysis of the key performance metrics of the proposed wearable antenna against existing designs. It evaluates parameters such as antenna dimensions, wavelength, SAR and designs to highlight the advantages and limitations of the proposed approach.

## 7. Performance characteristics comparison

The significant factors for performing the performance comparison between wearable antennas are input reflection coefficient, gain, directivity, SAR, bandwidth radiation efficiency, flexibility, and material properties. The section provides the performance analysis of different wearable antennae, including microstrip patch, monopole, slot, and textile-based antennas. Table 6 represents the performance characteristics of different antennas and the triangular-slotted wearable textile antenna. Here, we observe that the performance characteristics of the other antennas are not correctly determined, the bandwidth and the efficiency are low, and the SAR value is calculated only for the breast phantom model. The researchers have not done the comparative analysis of the flat and breast phantom correctly. However, the proposed antenna's performance characteristics have been determined correctly. The SAR value for the flat and breast phantom models is calculated and compared appropriately. The antenna exhibits a good frequency range, which is suitable for many BCWC, biotelemetry, and healthcare monitoring applications, and it has a good range and SAR value for early breast cancer detection.

**Table 6:** Performance comparison between the presented and other antennas.

References	Dimensions in λ^2^	Frequency Range (GHz)	SAR (W/Kg)	Design/Technique	Material (ℇ_r_)
[5]		1.86–2.4	0.58	Monopole	Cotton (2.8)
[9]	0.67λ × 0.67 λ	2.5, 3.5, 5.5	–	Triple Band	Felt (1.6)
[10]	0.43λ × 0.43 λ	2.4	2.70, 0.013	EBG (Electromagnetic Band Gap)	Denim (1.7)
[15]	0.57λ × 0.57 λ	2.85–9.81	1.3	–	Denim (1.7)
[13]	0.29λ × 0.29 λ	2.4, 5, 6	–	T-Shape	Polypropylene (2.2)
[18]	0.04λ × 0.02 λ	1.198–4.055	–	Compact	Cotton
[22]	0.43λ × 0.50 λ	2.45	0.52	EBG	Denim (1.6)
[26]	0.12λ × 0.27λ	2 - 6.23	2.45	MIMO (Multiple Input Multiple Output)	Felt (1.34)
[29]	0.20λ × 0.38 λ	2.4	–	–	Jeans (1.7)
Proposed	0.53λ × 0.60 λ	2.95–24.2	0.712 (Flat), 0.152 (Breast)	Circular, Triangular Slotted	Jeans (1.7)

## 8. Conclusion

In conclusion, the proposed triangular slotted circular UWB flexible antenna performs well for health care applications and is highly suitable for breast cancer detection. A brief review of research papers based on the recent approaches to designing microstrip patch antennas for breast cancer detection suggests that microwave imaging is a promising candidate for offering an alternative way of breast screening. Microwave exposure is considered safe for humans. Microwave imaging is considered a safer, low-cost alternative for tumor screening in the breast than other currently existing methods. In addition, wearable and non-wearable microstrip patch antennas have been surveyed for breast cancer detection and other on-body applications. The presented antenna is constructed from the jeans substrate having dielectric constant ℇr = 1.7, which is in between the ground and the patch layer, forms with the adhesive copper sheet with an ultra-wideband operation from 2.95 GHz to 24.2 GHz and large bandwidth of up to 156%; it can achieve around −37dB input reflection coefficient at the resonance frequency14.35GHz, which is very stable across operating range effects due its fractal radiator design. With a footprint measuring just 46.3 x 52.6 mm² and only 1.076 mm high, it is ideally fitted for use in wearable applications with safe specific absorption rate (SAR) values of the antenna are also achieved, with 0.712 W/kg for a flat phantom for 10 gm of tissue at an operating frequency and 0.152 W/kg in case of a breast phantom again for 10 gm of tissue, making it safe for deploying inside medical environments as well. The achievement of miniaturization, wide frequency range, and high bandwidth property suits it well for amplifying continuous health monitoring and breast cancer detection techniques for future trends.
